# Causal relationship between hypothyroidism and temporomandibular disorders: evidence from complementary genetic methods

**DOI:** 10.1186/s12903-024-03999-z

**Published:** 2024-02-17

**Authors:** Xin Chen, Junyu Xu, Zheng Cheng, Qianyi Wang, Zhibai Zhao, Qianglin Jiang

**Affiliations:** 1 Department of Oral and Maxillofacial Surgery, Jiangyin People’s Hospital Affiliated to Nantong University, No.163, Shoushan Road, Jiangyin, Jiangsu Province 214400 China; 2 Department of Cardiology, Jiangyin People’s Hospital Affiliated to Nantong University, No.163, Shoushan Road, Jiangyin, Jiangsu Province 214400 China; 3 Department of Oral Mucosal Diseases, The Affiliated Stomatological Hospital of Nanjing Medical University, Nanjing, China; 4 Department of Periodontics, Jiangyin People’s Hospital Affiliated to Nantong University, No.163, Shoushan Road, Jiangyin, Jiangsu Province 214400 China

**Keywords:** Thyroid, Hypothyroidism, Temporomandibular disorders, Mendelian randomization analysis, Causality

## Abstract

**Background:**

The role of thyroid health in temporomandibular disorders (TMDs) has been emphasized in observational studies. However, whether the causation exists is unclear, and controversy remains about which specific disorder, such as hypothyroidism or hyperthyroidism, is destructive in TMDs. This study aims to investigate the overall and specific causal effects of various thyroid conditions on TMDs.

**Methods:**

Mendelian randomization (MR) studies were performed using genetic instruments for thyrotropin (TSH, *N* = 119,715), free thyroxine (fT4, *N* = 49,269), hypothyroidism (*N* = 410,141), hyperthyroidism (*N* = 460,499), and TMDs (*N* = 211,023). We assessed the overall effect of each thyroid factor via inverse-variance weighted (IVW), weighted median, and MR-Egger methods, and performed extensive sensitivity analyses. Additionally, multivariable MR was conducted to evaluate the direct or indirect effects of hypothyroidism on TMDs whilst accounting for TSH, fT4 and hyperthyroidism, and vice versa.

**Results:**

Univariable MR analyses revealed a causal effect of hypothyroidism on an increased risk of TMDs (IVW OR: 1.12, 95% CI: 1.05–1.20, *p* = 0.001). No significant association between genetically predicted hyperthyroidism, TSH, or fT4 and TMDs. In the multivariable MR analyses, the effects of hypothyroidism on TMDs occurrence remained significant even after adjSusting for TSH, fT4 and hyperthyroidism (multivariable IVW OR: 1.10, 95% CI: 1.03–1.17, *p =* 0.006). No pleiotropy and heterogeneity were detected in the analyses (*p* > 0.05).

**Conclusions:**

Hypothyroidism might causally increase the risk of TMDs through a direct pathway, highlighting the critical role of managing thyroid health in the prevention of TMDs. Clinicians should give heightened attention to patients with hypothyroidism when seeking medical advice for temporomandibular discomfort. However, caution is warranted due to the potential confounders, pleiotropy, and selection bias in the MR study.

**Supplementary Information:**

The online version contains supplementary material available at 10.1186/s12903-024-03999-z.

## Background

Temporomandibular disorders (TMDs) constitute a diverse array of musculoskeletal and neuromuscular conditions affecting the temporomandibular joints (TMJ), surrounding musculature, and osseous structures. Common symptoms and signs include preauricular pain, restricted or deviating range of motion, and joint sounds, profoundly impacting individuals’ quality of life [1]. The recent Diagnostic Criteria for Temporomandibular Disorders (DC/TMD) classify these conditions into four primary categories: intra-articular and extra-articular disorders, headache attributed to TMDs, and disorders involving associated structures [2]. Epidemiological studies have revealed that TMDs affect 5–12% of adults, with an annual financial cost exceeding 100 billion dollars in the United States [3, 4]. Despite the rising burden, the licensed treatment of TMDs can just alleviate the symptoms [5]. The multifaceted etiology of TMDs includes socioeconomic factors, emotional influences, oral parafunctions, and trauma events. Recent observational research suggests systemic disorders or diseases (e.g., COVID-19 infection, estrogen levels, diabetes) may contribute to TMDs occurrence by fostering inflammation and disturbing bone metabolism in the TMJ [6–8]. Therefore, understanding the pathogenesis is urgently required for the development of disease-modifying treatments for TMDs.

Observational studies have also underscored the pivotal role of thyroid hormone in bone disorders, including TMDs [9–11]. Thyroid dysfunction encompassed two main conditions: hypothyroidism, characterized by underproduction of thyroid hormones, and hyperthyroidism, marked by excess production [9]. In the fifth Korea National Health and Nutrition Examination Survey, 14% of individuals with thyroid dysfunction were found to have TMDs [12]. However, the correlation between thyroid health and TMDs remains a subject of debate. In a cohort study involving 712 patients with thyroid dysfunction and a matched number of healthy adults, no significant difference in TMDs incidence was observed [12]. Subsequent observational studies, after adjusting for economic and lifestyle factors, indicated a positive association between thyroid dysfunction, particularly hypothyroidism, and TMDs [10, 12]. Additionally, TMDs exhibit a high comorbidity rate with Hashimoto’s thyroiditis, the leading cause of hypothyroidism [10]. Shared symptoms like psychological disorders, muscle pain and weakness are evident in both TMDs and thyroid dysfunction [5, 10]. Furthermore, in vivo studies, machine learning analyses, and genetic investigations have provided supporting evidence for thyroid dysfunctions as a risk factor for TMDs [4, 13, 14]. Nevertheless, establishing a definitive causal relationship between them remains challenging due to potential biases, confounding factors, and the possibility of reverse causality in these observational studies [10, 12].

Mendelian randomization (MR) employs genetic variation as an instrumental variable to infer causality [15]. By adhering to Mendel’s second law, genetic variation is randomly assigned during fertilization, reducing the impact of confounding factors and approximating the effect of randomized controlled trials [16]. MR also avoids issues of reverse causation, as genetic variation remains unaffected by disease status. Previous MR studies have demonstrated causal relationships between thyroid dysfunction and various joint conditions, including rheumatoid arthritis, hallux valgus, and lower bone mineral density [17–19]. Recent releases of genome-wide association study (GWAS) data for hypothyroidism and hyperthyroidism have provided robust genetic instruments for MR analysis, addressing concerns about weak-instrument bias [20]. To comprehensively evaluate the relationship between thyroid health and TMDs, it is crucial to incorporate an assessment of other thyroid indicators, such as thyroid-stimulating hormone (TSH) and thyroid hormone levels [15]. Furthermore, considering the genetic interconnections among various thyroid disorders, it becomes important to investigate their specific effects on the risk of developing TMDs.

Given the limited understanding of the causal effects of thyroid health on TMDs, this study employed GWAS statistics to systematically assess the potential causalities using univariable and multivariable MR methods. Firstly, we utilized human genetic data within the MR framework to probe the overall causal link between thyroid diseases or hormones and the risk of TMDs. Secondly, we quantified the specific effects of significant indicators on the likelihood of developing TMDs. Our findings not only elucidate the causal relationship between thyroid health and TMDs, but also support a direct effect of hypothyroidism on TMDs, providing a novel strategy for the clinical intervention.

## Methods

### Study design

**Fig. 1:**
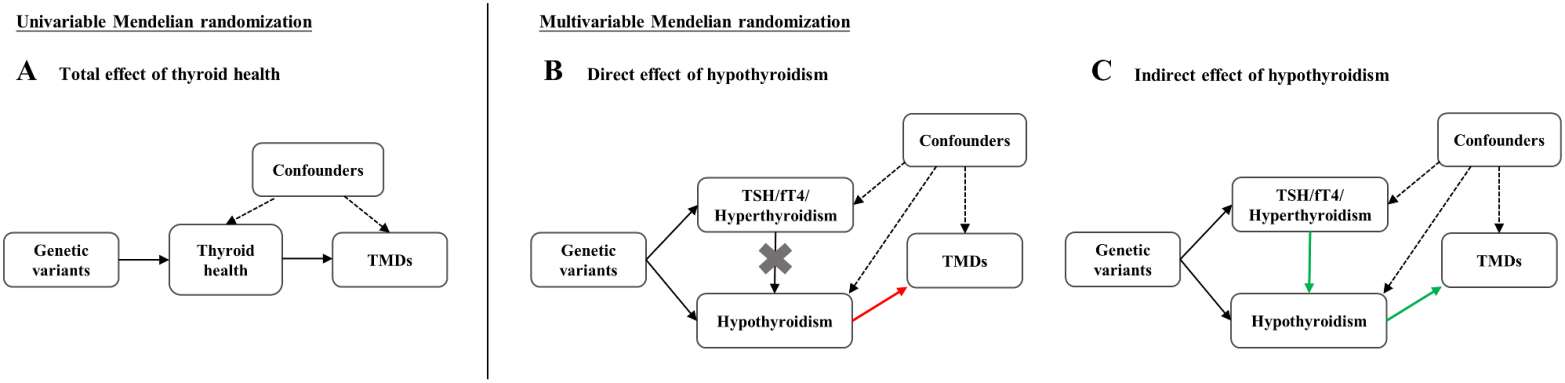
Overview of the present MR study. (**A**) Univariable MR analyses to estimate overall effects between thyroid health (hypothyroidism; hyperthyroidism; thyroid-stimulating hormone, TSH; free thyroxine, fT4) and temporomandibular disorders (TMDs); (**B**) Multivariable MR analyses to estimate direct effects of hypothyroidism of TMDs; (**C**) Applying the same multivariable framework to estimate the indirect effects on TMDs mediated along the causal pathway via TSH, fT4 or hyperthyroidism. The colored arrows in red and green on these graphs illustrate the causal effect of hypothyroidism on the outcome being estimated in multivariable MR analyses

Figure 1 illustrates the study design for MR. To satisfy the three core assumptions of MR, genetic variants should meet specific criteria. Firstly, they should demonstrate a strong correlation with the exposure. Secondly, they are independent of confounding factors associated with both the exposure and outcome. Thirdly, genetic variants influence outcomes only through the exposure. We sourced genetic information for exposures from reputable GWAS consortia, and the validity of instrumental variables was confirmed in original studies [20–22], thereby satisfying the first assumption. The latter two assumptions collectively constitute the independence of horizontal pleiotropy, which could be indirectly assessed using various statistical methods. The abbreviations used in the text is listed in Table 1.

**Table 1:** The list of abbreviations used in the text

Abbreviation	Definition
TMDs	Temporomandibular disorders
MR	Mendelian randomization
GWAS	Genome-wide association studies
TSH	Thyroid-stimulating hormone
fT4	Free thyroxine
SNPs	Single nucleotide polymorphisms
IVW	Inverse-variance weighted
MR-PRESSO	Mendelian randomized polymorphism RESidual Sum and Outlier
LOO	Leave-one-out
T3	3,5,3’-L-triiodothyronine

### Participants and data sources

For this study, we selected TSH, free thyroxine (fT4), hypothyroidism and hyperthyroidism as indicators for thyroid health (Supplementary Table 1). It is important to note that the TSH and fT4 levels of included individuals fell within the cohort-specific reference ranges. Additionally, individuals of non-European ancestry, those using thyroid medication (defined as Anatomical Therapeutic Chemical code H03), those with values outside the reference range, those with any thyroid disorders (based on the ICD9 and ICD10 codes mapped to PheCodes 193, 244, 245 and 246), or those with history of thyroid surgery were excluded in TSH and fT4 GWAS [20, 22]. Summary-level data for TSH (*N* = 119,715) was derived from meta-analyses conducted by the ThyroidOmics consortium (*N* = 54,288), the Michigan Genomic Initiative (*N* = 10, 085) and the Nord-Trøndelag Health Study (*N* = 55,342) [22]. Similarly, the GWAS data for fT4 were collected from 49,269 individuals in the ThyroidOmics consortium, representing the largest GWAS dataset for thyroid function to date [20]. Publicly accessible summary statistics data for hypothyroidism (*N* = 410,141) and hyperthyroidism (*N* = 460,499) were obtained from the IEU OpenGWAS project website (https://gwas.mrcieu.ac.uk). Hypothyroidism cases were defined using the revised International Classification of Diseases (ICD-10) codes E03 (case *N* = 30,155) and remaining subjects were taken as controls (*N* = 379,986), excluding those related to iodine-deficiency-related or postprocedural hypothyroidism [21]. Hyperthyroidism was defined according to the ICD-10 codes E05 (cases *N* = 3,557) [21]. Participants experiencing chronic thyroiditis with transient thyrotoxicosis or neonatal thyrotoxicosis were excluded and remaining subjects were taken as controls (*N* = 456,942). More detailed information on participants selection and data processing can be found in original articles.

The most recent dataset on TMDs was obtained from the FinnGen project. TMDs cases were defined using the ICD-10 codes K07.60, K07.61, K07.62, and K07.63 (https://risteys.finngen.fi/endpoints/TEMPOROMANDIB). Excluding individuals with painful conditions affecting the limbs, back, neck, and abdomen, a total of 5,668 cases of TMDs, along with 205,355 controls, were acquired from the GWAS data for the investigation. All GWAS analyses in this study were exclusively conducted on populations of European descent, ensuring that the necessary ethical approvals and participant consents were diligently obtained.

### Instruments selection

We followed a rigorous selection procedure in line with previous MR studies [23, 24]. Initially, we identified single nucleotide polymorphisms (SNPs) significantly associated with the exposure (*p* < 5 × 10^− 8^). Instrument variables were subsequently clumped, retaining only independent SNPs (r^2^ ≥ 0.001, clumping window ≤ 10,000 kb) with the lowest p-value. Meanwhile, we utilized the PhenoScanner V2 website (http://www.phenoscanner.medschl.cam.ac.uk) to exclude SNPs associated with potential confounders such as pain, smoking, or psychosocial conditions, as well as the outcome of interest [9, 23]. Next, we extracted the corresponding SNPs from the outcome dataset and removed those showing a significant association with the outcome (*p* < 5 × 10^− 8^). For SNPs absent in the outcome dataset, suitable proxies with high linkage disequilibrium (r^2^ > 0.8) were selected. In cases where no appropriate proxies could be identified, we excluded those SNPs from further analysis. Finally, we conducted harmonization on the remaining exposure and outcome SNPs to ensure compatibility and removed any ambiguous or incompatible SNPs. R^2^ and F statistics were calculated as previously described, with an F statistic > 10 typically considered indicative of strong instrument strength [25].

### Statistical analysis

To accurately examine causal relationship between thyroid health and TMDs, we employed several complementary methods of MR, including inverse-variance weighted (IVW), weighted median, and MR Egger regression. The primary analysis, conducted using IVW, combines the Wald ratios for each SNP to obtain a pooled estimate. Sensitivity analysis played a pivotal role in identifying underlying pleiotropy and heterogeneity in MR estimates. We assessed heterogeneity among different genetic variations using Cochran’s Q test. Horizontal pleiotropy was detected through the MR-Egger intercept test, Mendelian randomized polymorphism RESidual Sum and Outlier (MR-PRESSO), and leave-one-out (LOO) analyses. If any outlier SNPs were identified using MR-PRESSO, we removed them and repeated the MR analysis.

Given the hypothesis that hypothyroidism, hyperthyroidism, TSH, and fT4 might act as confounders to each other and bring interference to the results of univariable MR analysis. We further conducted a multivariable MR analysis using the IVW method, MR-Egger regression, and median method. These comprehensive approaches enabled us to estimate the direct or indirect effect of hypothyroidism on the risk of TMDs while accounting for hyperthyroidism, TSH, fT4, and vice versa. Furthermore, the p-value of MR-Egger intercept serves as an indicator of horizontal pleiotropy.

In the univariable MR analysis, four traits representing thyroid health were included. To account for multiple testing, we applied a Bonferroni correction and set the threshold for statistical significance at a p-value of 0.013 (0.05/4). Associations with p-values between 0.013 and 0.050 were considered suggestive evidence of an association. All MR analyses were performed using R (version 4.3.0) through the TwoSampleMR package (version 0.5.6), MRPRESSO (version 1.0), and MendelianRandomization (version 0.7.0).

## Results

Detailed information on the SNPs for each thyroid indicator, including all characteristics following the initial screening process, elimination of confounders (such as pain, smoking, nerve-related conditions, anxiety, and tension), and data harmonization, can be found in Supplementary Tables 2–4. Specifically, we identified a total of 67 independent SNPs associated with TSH, 16 SNPs for fT4, 54 SNPs for hypothyroidism, and 8 SNPs for hyperthyroidism (Supplementary Table 4). The F-statistics of the genetic instruments for thyroid health ranged from 30.0 to 1231.2, indicating no substantial evidence of weak instrument bias.

**Fig. 2:**
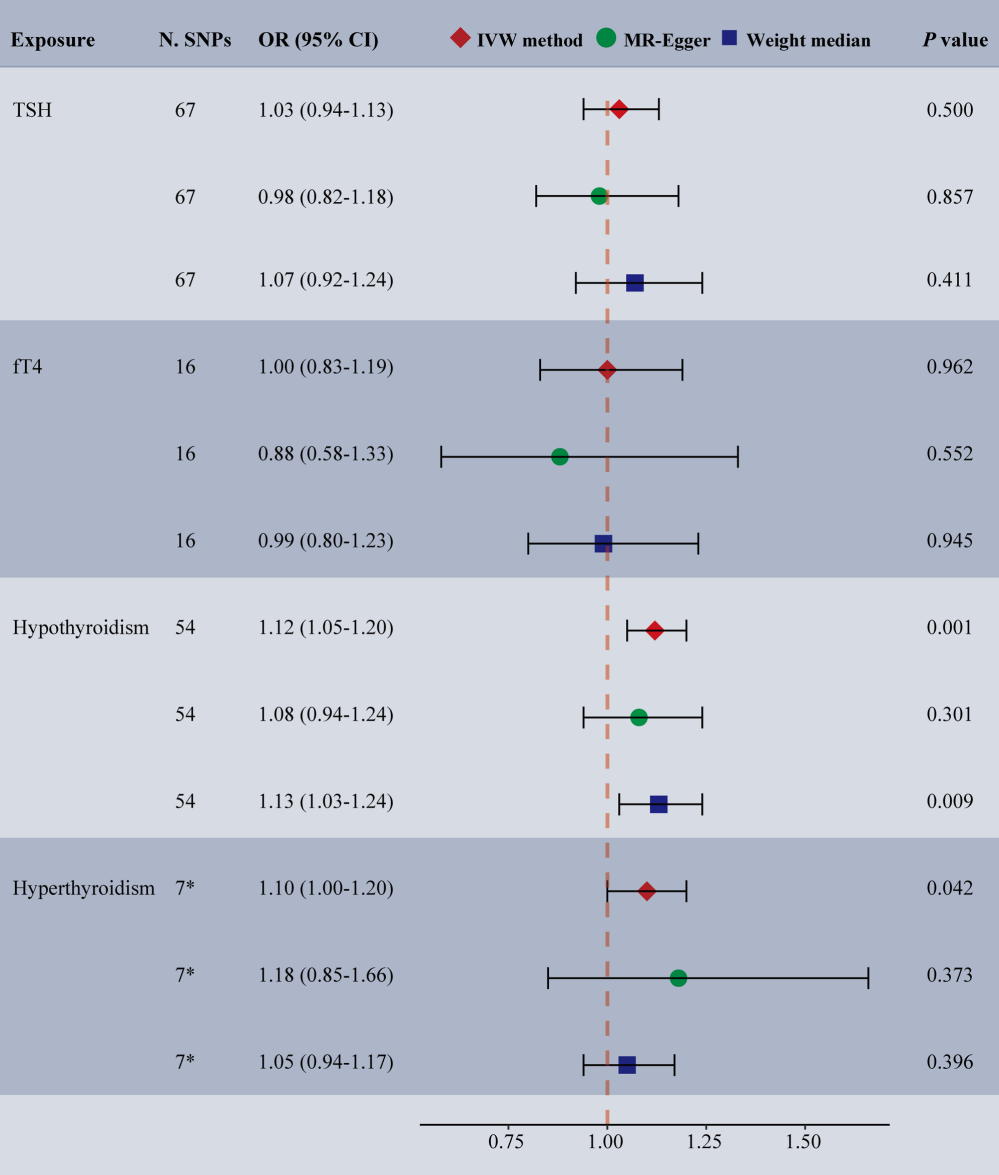
Forest plot depicting MR results for the association of genetically proxied thyroid health with temporomandibular disorders. *Abbreviations* N. SNPs, number of SNPs used in MR; OR, odds ratio; CI, confidence intervals; IVW, inverse variance weighted; TSH, thyroid-stimulating hormone; fT4, free thyroxine. * The results after excluding the outlier. The results before correction were shown in Supplementary Table 5

**Fig. 3:**
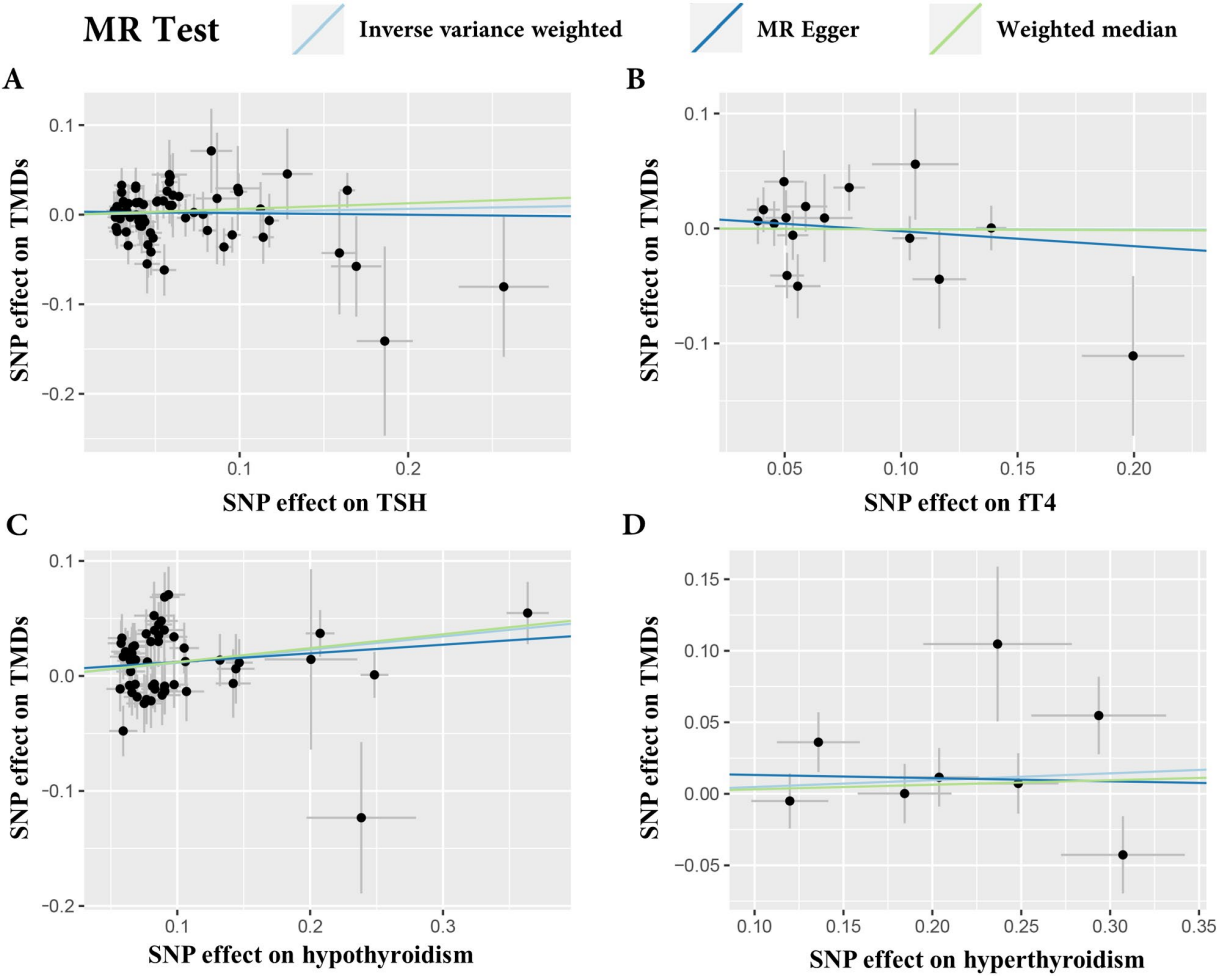
Scatter plot of the causal relationship between thyroid health and temporomandibular disorders using different MR methods. (**A**) Causal estimates for thyroid-stimulating hormone (TSH) on temporomandibular disorders (TMDs); (**B**) Causal estimates for free thyroxine (fT4) on TMDs; (**C**) Causal estimates for hypothyroidism on TMDs; (**D**) Causal estimates for hyperthyroidism on TMDs

Among the examined phenotypes, the IVW analysis revealed a positive causal relationship between hypothyroidism and TMDs (OR: 1.12, 95% CI: 1.05–1.20, *p* = 0.001) (Fig. 2). The weighted median analysis provided similar causal estimates for hypothyroidism (OR: 1.13, 95% CI: 1.03–1.24, *p* = 0.009), while MR Egger analysis did not yield significant results (OR: 1.08, 95% CI: 0.94–1.24, *p* = 0.301). However, no evidence of a causal association was observed between hyperthyroidism, TSH, fT4, and the onset of TMDs (*p* > 0.05) (Fig. 2, Supplementary Table 5). MR-PRESSO identified one SNP outlier (rs758778) when evaluating the effects of hyperthyroidism on TMDs (*p* of global test = 0.039). After excluding the outlier, genetically proxied hyperthyroidism showed a dominant but non-significant association with TMDs (IVW OR: 1.10, 95% CI: 1.00-1.20, *p* = 0.042) (Fig. 2). No evidence of heterogeneity and horizontal pleiotropy were observed (*p* > 0.05) (Table 2; Fig. 3). Furthermore, the LOO analysis did not identify any SNP strongly deviating from the overall effect of thyroid health on TMDs (Supplementary Fig. 1). The funnel plots exhibited asymmetry, indicating no pleiotropy (Supplementary Fig. 2).

**Table 2:** Sensitivity analysis of the associations between thyroid health and temporomandibular disorders

Exposure	Cochran Q test				MR-Egger			MR-PRESSO
	Q value	I^2^	P		Intercept	P		P of global test
TSH	59.405	0.111	0.704		0.003	0.548		0.823
fT4	19.915	0.247	0.175		0.011	0.524		0.062
Hypothyroidism	70.167	0.245	0.057		0.005	0.542		0.116
Hyperthyroidism	6.688	0.103	0.351		-0.015	0.668		0.267

**Fig. 4:**
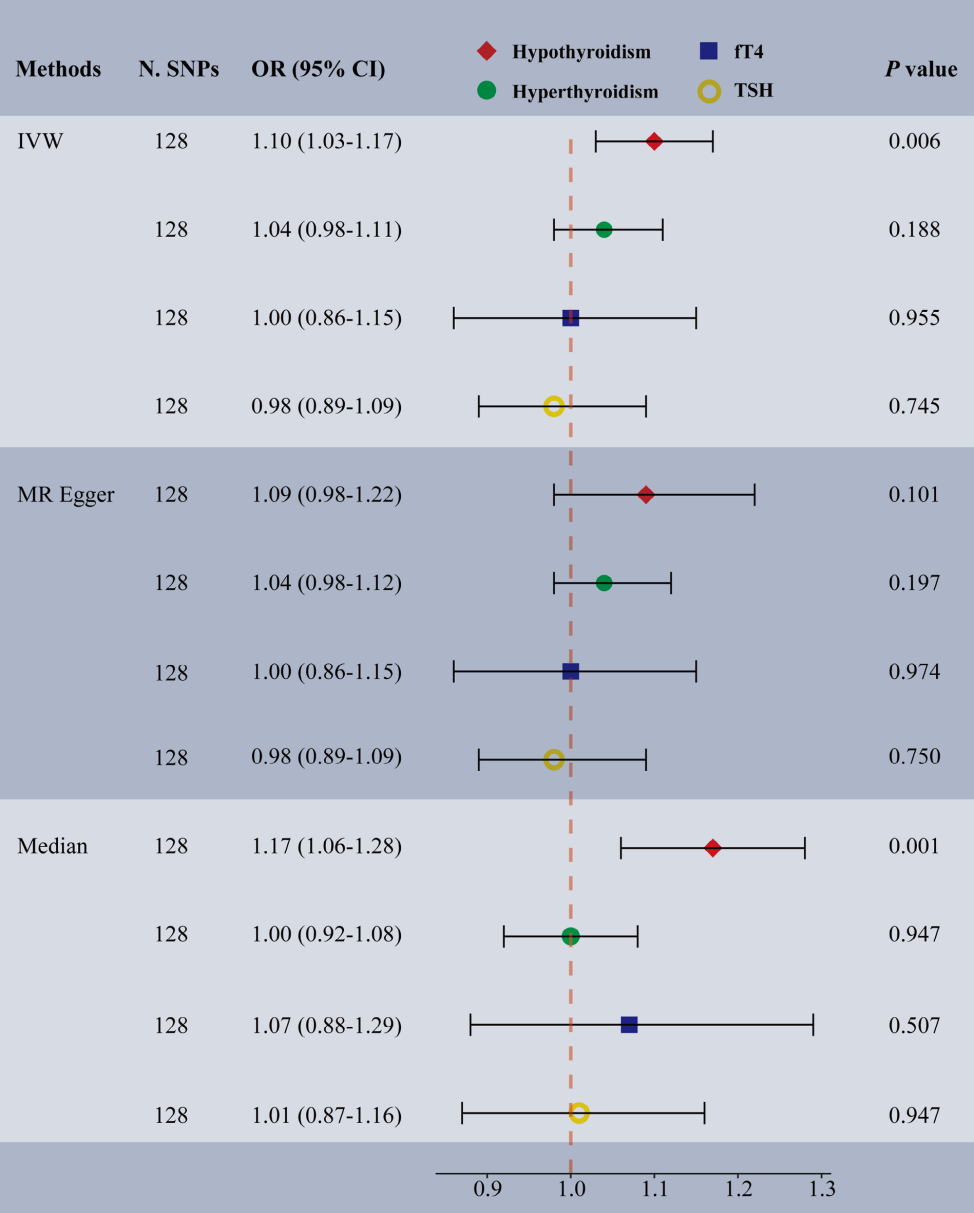
Multivariable MR estimating the association of thyroid-stimulating hormone, free thyroxine, hypothyroidism, and hyperthyroidism with temporomandibular disorders. Red plots represent the multivariable MR analyses of the effects of hypothyroidism on temporomandibular disorders (TMDs) after adjusting for hyperthyroidism, thyroid-stimulating hormone (TSH), and free thyroxine (fT4); Green plots represent the multivariable MR analyses of the effects of hyperthyroidism on TMDs after adjusting for hypothyroidism, TSH, and fT4; Blue plots represent the multivariable MR analyses of the effects of the effects of fT4 on TMDs after adjusting for hypothyroidism, TSH, and hyperthyroidism; Yellow plots represent the multivariable MR analyses of the effects of the effects of TSH on TMDs after adjusting for hypothyroidism, fT4, and hyperthyroidism; Abbreviations: N. SNPs, number of SNPs used in MR; OR, odds ratio; CI, confidence intervals; IVW, inverse variance weighted

In the multivariable IVW analyses, we discovered a significant and direct effect of hypothyroidism on the risk of TMDs (OR: 1.10, 95% CI: 1.03–1.17, *p =* 0.006) (Fig. 4). However, there was limited evidence of a direct or indirect association between TMDs and genetically predicted TSH (OR: 0.98, 95% CI: 0.89–1.09, *p =* 0.745), fT4 (OR: 1.00, 95% CI: 0.86–1.15, *p =* 0.955), or hyperthyroidism (OR: 1.04, 95% CI: 0.98–1.11, *p =* 0.188). The Cochran’s Q test indicated that the effect estimates from all instrumental variables were not heterogenous (Supplementary Table 6). Additionally, the multivariable median analyses provided consistent results for hypothyroidism (OR: 1.17, 95% CI: 1.06–1.28, *p =* 0.001), hyperthyroidism (OR: 1.00, 95% CI: 0.92–1.08, *p =* 0.947), fT4 (OR: 1.07, 95% CI: 0.88–1.29, *p =* 0.507) and TSH (OR: 1.01, 95% CI: 0.87–1.16, *p =* 0.947). Moreover, the multivariable MR-Egger analyses provided minimal evidence of pleiotropy (intercept < 0.001, *p =* 0.902).

## Discussion

To date, this is the first study to investigate the causal link between thyroid health and TMDs, employing various complementary MR approaches. Based on univariable MR analysis, there was evidence suggesting that genetically predicted hypothyroidism exerts a total effect on TMDs risk. Furthermore, when accounting for TSH, fT4, and hyperthyroidism, the effects of hypothyroidism on TMDs were primarily found to be direct. Meanwhile, there was little proof for the function of hyperthyroidism, TSH, or fT4 in causing TMDs.

Currently, a series of epidemiologic studies have demonstrated a strong association between thyroid health and TMDs. According to one prevailing theory, the imbalance in bone development and remolding caused by thyroid dysfunction may contribute to the pathological process of TMDs [11]. In a comprehensive cross-sectional study involving 25,534 adults, positive associations between TMDs and chronic diseases were revealed [12]. Further multivariate logistic regression analyses indicated thyroid dysfunction as a risk factor for TMDs (OR: 1.49, 95%CI: 1.13–1.96), and a U-shaped relationship between TSH and TMDs was observed. Recently, a single-center prospective case-control study also demonstrated that patients with Hashimoto’s thyroiditis had a significant risk of TMDs, particularly in cases of disc displacement with reposition, muscle pain, and stiffness [10]. The adverse psychological factors induced by thyroid dysfunction, such as depression, anxiety, and nervousness, were suggested to play a crucial role in the development of TMDs [10]. However, these results may be limited by several factors, such as different diagnostic methods, insufficient samples, a relatively short study duration, and limited portion of TMDs cases.

Our findings provide a new perspective on the etiology of TMDs, indicating that hypothyroidism, rather than hyperthyroidism, may contribute to their onset. In vivo studies in rats or zebrafish with induced thyroid dysfunction support this notion, demonstrating that hypothyroidism can lead to varying degrees of temporomandibular dysplasia [13, 14]. This is characterized by reduced opening degree, delayed jaw ossification, and a shorter anguloarticular relative to the coronoid process [26]. The positive association between hypothyroidism and TMDs is biologically plausible. Firstly, the skeleton is a 3,5,3’-L-triiodothyronine(T3)-target organ, and hypothyroidism could delay signaling transportation [9]. In this condition, despite maximum type 2 deiodinase (DIO2) and minimum type 3 deiodinase (DIO3) activities, thyroid hormone receptors TRα1 remains unliganded and bound to corepressor, thus inhibiting T3-target gene transcription in bone cells. In hyperthyroidism, despite maximum DIO3 and minimum DIO2 activities, supraphysiological intracellular T3 concentrations result in increased TRα1 activation and enhanced gene responses. Secondly, the temporomandibular joint or disk is more susceptible to displacement due to the reduction in masticatory muscle strength and tenderness associated with hypothyroidism [12]. The incidence of myopathies in hypothyroidism ranges from 30 to 80%, with typical symptoms such as weakness, muscular scamps, and myalgia [10, 27, 28]. Thirdly, the genetic instrument selected for hyperthyroidism in existing research only included a limited set of genetic markers [19, 25, 29]. It is conceivable that certain aspects of hyperthyroidism, not captured by these specific SNPs, could potentially introduce information bias. Fourthly, thyroid hormones are vital regulators of bone maturation, energy metabolism, cellular turnover, and craniofacial development. Recent in vivo research by Gecgelen et al. has suggested that untreated hypothyroidism may contribute to increased hyperdivergent facial dimensions in rat pups [14]. Furthermore, statistically thicker cortical bone was observed in hyperdivergent patients compared to normal-divergent and hypodivergent patients in CBCT measurements [30]. It could be hypothesized that individuals with hypothyroidism may present with a high-angle mandibular plane, leading to higher loads and increased frequency of traumatic events in the TMJ. Further investigation is needed to determine whether the vertical skeletal pattern could serve as a mediator between hypothyroidism and TMDs. Fifthly, hypothyroidism is suggested to have detrimental effects on synovial tissue and chondrocytes, compromising the joints’ remodeling ability. For instance, musculoskeletal ultrasound abnormalities were more commonly associated with hypothyroid states compared to euthyroid states [31]. Animal studies have indicated that elevated TSH levels (10mU/mL) could inhibit the proliferation and autophagy levels of primary mouse chondrocytes (PMCs), as well as induce apoptosis of PMCs via the BAX/Bcl-2 pathway, ultimately leading to a significant reduction in cartilage cellularity within hypothyroid condyles and an overexpression of matrix metalloproteinases [32–34].

Moreover, studies using continuous exposures, including TSH and fT4, are considered necessary. As a sensitive marker for thyroid function, TSH could reflect both hypothyroidism and subclinical hyperthyroidism [35]. However, the data showed a negative causal relationship between fT4 and TMDs, as well as a positive association between TSH and TMDs, though neither reached statistical significance. These non-significant results seemed to align with the role of an underactive thyroid in TMDs development [10]. There were several potential reasons for these outcomes. Firstly, the dataset for TSH and fT4 included multiple cohorts, and the values in each cohort were derived from respective normal ranges [20, 22]. However, in cases of hypothyroidism or hyperthyroidism, TSH and fT4 levels may fall outside of these ranges. Secondly, thyroid signaling can be customized at the cellular level through regulation of thyroid hormone transporters, deiodinases, and nuclear thyroid hormone receptors among others [9]. The intracellular exposure to T3 is thus somewhat independent of centrally regulated TSH and fT4, which is especially important in orchestrating proliferation and differentiation of stem cells and progenitor cells. To the best of our knowledge, no GWAS of T3 level has been published to date, preventing us from validating this mechanism using the MR approach.

Although previously reported associations could be influenced by unadjusted confounders, bidirectional causality in observational studies, our MR research supported that hypothyroidism and TMDs are causally related. It is important to acknowledge that the IVW approach used in MR assumes the validity of all genetic variants and may be susceptible to bias from pleiotropic effects [36]. However, to mitigate these limitations, we employed the weighted median method, allowing for consistent estimates even when at least half of the instruments are potentially invalid [37]. Additionally, we utilized the MR-Egger method, which enables analysis in the presence of all invalid instruments [38]. The MR PRESSO method was applied to detect and correct for any outlier SNP reflecting likely horizontal pleiotropic biases for MR causal estimates [39]. Importantly, almost all MR approaches yielded consistent results, indicating the robustness of our findings. Furthermore, complementary statistical methods did not identify dominant evidence of heterogeneity or horizontal pleiotropy in the MR study.

Managing both TMDs and thyroid disorders necessitates a multidisciplinary approach. Clinical observations have shown that various factors, such as injury, arthritis, and psychological elements, may play an important role in the progression of TMDs and associated degenerative alterations [1]. Our study provides novel and valuable insights for the prevention and treatment of TMDs, particularly in highlighting adults with hypothyroidism as a potential high-risk group. Consequently, each case of TMDs must be approached individually for diagnosis and subsequent treatment. We recommend regular follow-up visits to monitor endocrine levels and jaw function, along with early treatments in patients with hypothyroidism. Furthermore, a multidisciplinary assessment of patients seeking dental care revealed concerning statistics. Among 379 participants, 29% were diagnosed with a chronic disease, and nearly 6% had thyroid dysfunction [40]. Given the significant impact of thyroid health, dentists should pay greater attention to endocrine and medication details, especially for those seeking advice on temporomandibular discomfort. Additionally, case reports suggested that simultaneous treatment of thyroiditis could alleviate pain and dysfunctional symptoms in both temporomandibular joints. Collaborative efforts among dentists, endocrinologists, and other healthcare professionals ensure holistic care, addressing the intricate relationship between hypothyroidism and the onset of TMDs.

## Limitations

However, caution should also be exercised when interpreting the findings of the present study, as there are several limitations that warrant consideration. Firstly, the effect estimates of the detected causal association between genetically determined hypothyroidism and TMDs was relatively modest. Furthermore, considering the potential causal effect of hyperthyroidism as estimated in the univariable MR analyses, the suggested U-shaped relationship between TSH and TMDs in the national epidemiological research may hold significant weight [12]. Secondly, our survey was exclusively conducted within a European population, limiting the generalization of our results due to potential ethnic and educational influences on causality. Thirdly, women in their middle age are most likely to develop TMDs and thyroid dysfunction [19, 41]. The participants in the thyroid GWAS had an average age about 55 years, with 53.8% being female, whereas those in the TMDs GWAS were on average 43 years old, with 75% being female [20–22]. While we initially sought age and gender-stratified GWAS data, none were available for thyroid health and TMDs. The lack of homogeneity in patient selection regarding age, sex, and potential concomitant pathologies may introduce bias into the findings. Variations in these demographic and clinical factors could confound the interpretation of results. Fourthly, the available GWAS globally classified TMDs, and the specific causal effect of hypothyroidism on subgroups of TMDs remains unknown. Further genetic studies should be conducted to differentiate the TMDs into articular and muscular conditions. Lastly, while the MR approach demonstrates remarkable performance in estimating causality, further research is needed to validate the impact of hypothyroidism on TMDs and to comprehend the underlying mechanisms.

## Conclusion

Our findings indicate that hypothyroidism causally increases the risk of TMDs through a direct pathway, highlighting the critical role of managing thyroid health in TMDs prevention. Clinicians should pay more attention to patients with thyroid dysfunction when seeking medical advice for temporomandibular discomfort. However, caution is warranted due to the potential confounders, pleiotropy, and selection bias in the MR study.

### Electronic supplementary material

Below is the link to the electronic supplementary material.

**Supplementary Material 1:** Supplementary Tables

**Supplementary Material 2:** Supplementary Figures

## Data Availability

All data generated or analyzed during this study are included in supplementary material or in the data repositories listed in the methods

## Abbreviations

MV-IVW: Multivariable inverse variance weighted
MV-Egger: Multivariable MR Egger
MV-Median: Multivariable median
OR: Odds ratio
CI: Confidence intervals
TSH: Thyroid-stimulating hormone
fT4: Free thyroxine
