# Supplementary material for: Causal relationship between hypothyroidism and temporomandibular disorders: evidence from complementary genetic methods
Source: BMC Oral Health. 2024 Feb 17;24:247. doi: 10.1186/s12903-024-03999-z (PMC10873979; doi:10.1186/s12903-024-03999-z)
Supplement: Supplementary file 2 — Supplementary Material 2: Supplementary Figures [file 12903_2024_3999_MOESM2_ESM.docx]

**Supplementary Fig. 1** Leave-one-out plots of estimates from genetically predicted thyroid health on temporomandibular disorders using different MR methods.


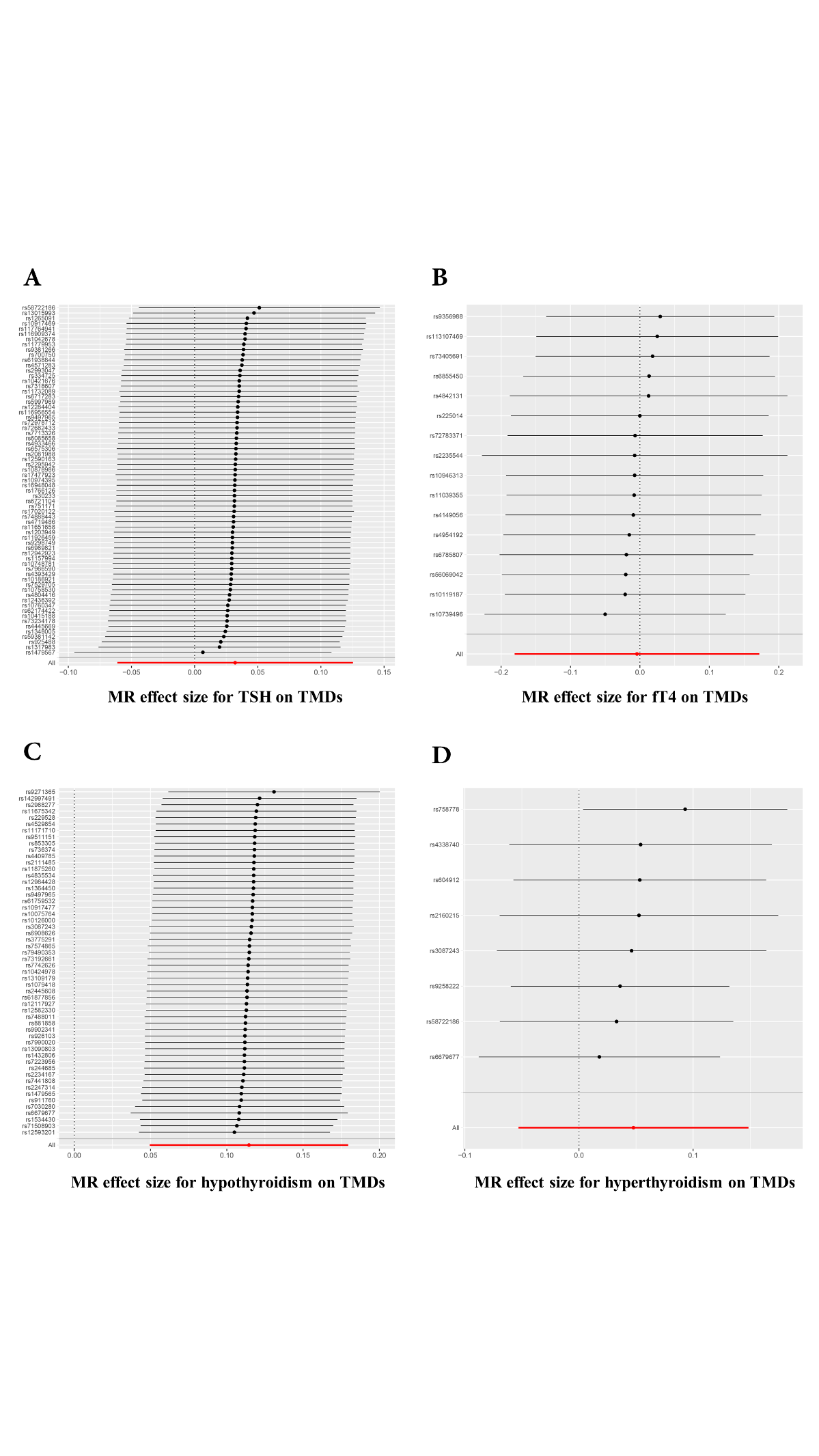


Leave-one-out plots of estimates from (A) genetically predicted thyroid-stimulating hormone (TSH), (B) free thyroxine (fT4), (C) hypothyroidism, (D) hyperthyroidism on temporomandibular disorders (TMDs).

**Supplementary Fig. 2** Funnel plots of estimates from genetically predicted thyroid health on temporomandibular disorders.


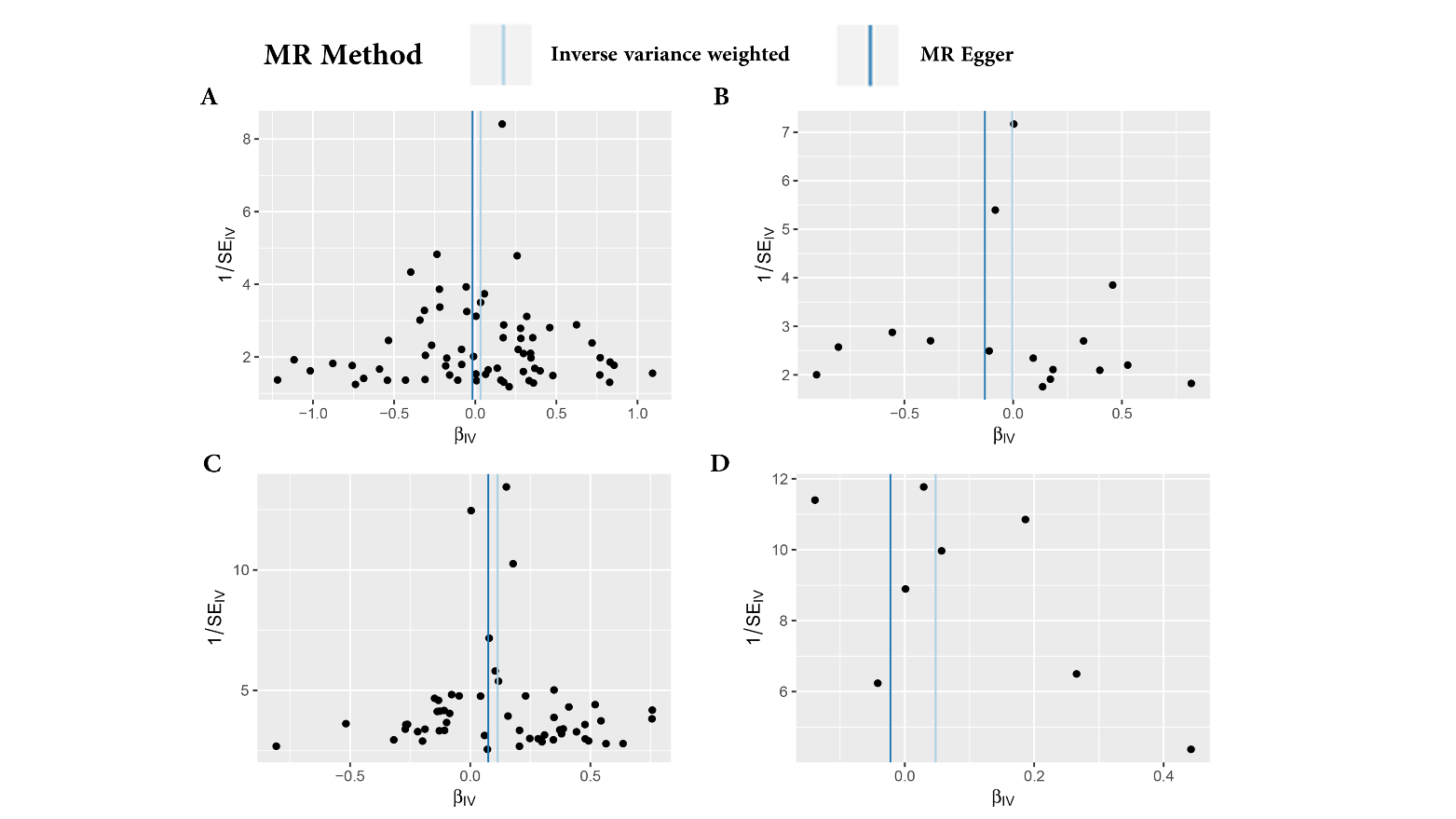


Funnel plots of estimates from (A) genetically predicted thyroid-stimulating hormone (TSH), (B) free thyroxine (fT4), (C) hypothyroidism, (D) hyperthyroidism on temporomandibular disorders.
